# Adsorption Behavior of CH_4_ Gas Molecule on the MoX_2_(S, Se, Te) Monolayer: The DFT Study

**DOI:** 10.1186/s11671-019-3125-5

**Published:** 2019-08-22

**Authors:** Jian Ren, Hui Liu, Yanyan Xue, Lin Wang

**Affiliations:** 1 School of Computer Science and Technology, Huaiyin Normal University, Chang Jiang West Road 111, Huaian, Jiangsu, 223300 China; 2 School of Physics and Optoelectronics, Xiangtan University, Yuhu District, Xiangtan, 411105 Hunan China

**Keywords:** CH_4_ gas molecule, Monolayer MoS_2_, Band gap, DFT, Charge transfer, Adsorption energy, Sensor

## Abstract

We predict the CH_4_-sensing performance of monolayer MoX_2_(S, Se, Te) with X-vacancy, Mo-vacancy, and divacancy by the density functional theory (DFT). The results demonstrate that the combination of different sixth main group elements with Mo atom has different adsorption behaviors for CH_4_ gas molecule. Compared with MoX_2_, MV_X_, MV_Mo_, and MV_D_ generally exhibit better adsorption properties under the same conditions. In addition, different defects will have different effects on adsorption behavior of the systems, the MV_D_(MoTe_2_) has the better adsorption, the better charge transfer, and the shortest distance in these systems. The results are proposed to predict the CH_4_ gas molecule adsorption properties of MV_D_(MoTe_2_) and would help in guiding experimentalists to develop better materials based on MoX_2_ for efficient gas detection or sensing applications.

## Introduction

**Fig.1:**
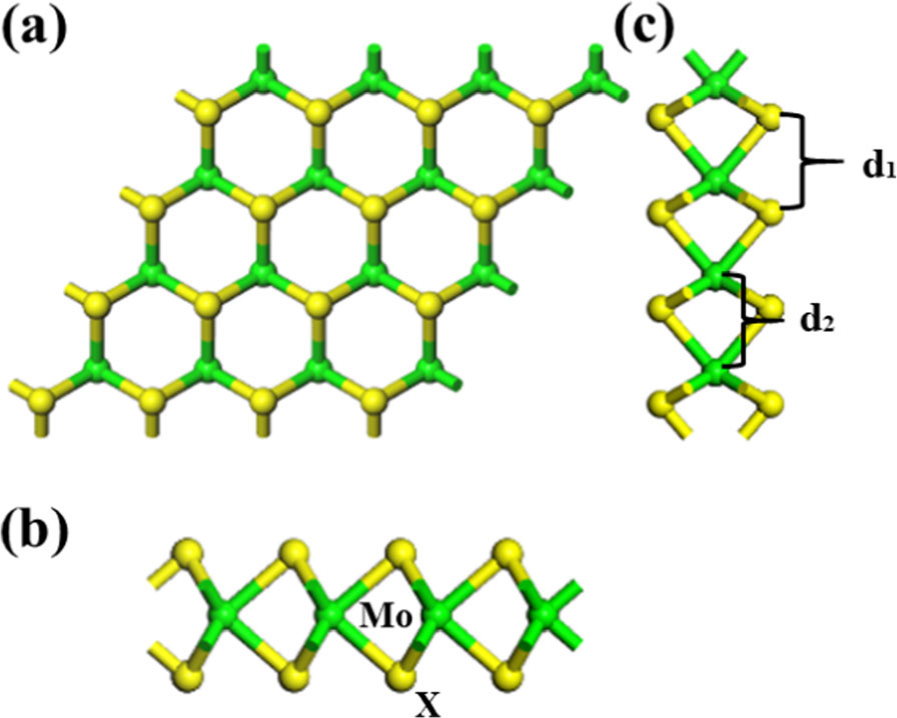
**a** Front view. **b** Side view. **c** Left view

Methane (CH_4_) is the simplest organic compound with colorless and tasteless gas [1–4], which is basically non-toxic to human beings, the oxygen content in the air will obviously decrease when the concentration of methane is too high, which makes people suffocate. When the concentration of methane reaches 25–30% in the air, it will cause headaches, dizziness, fatigue, inattention, rapid breathing and heartbeat, and ataxia [5–7]. Since the rise of graphene [8, 9] and the discovery of topological insulators [10], a lot of interesting physics have been found in systems with reduced dimensions. Other two-dimensional (2D) material, such as monolayers or few-layer systems (nanolayers) of transition-metal dichalcogenides (TMDs), gain importance because of their intrinsic band gap [11–15]. TMDs are MX_2_-type compounds where *r*(S, Se, Te) [16–19]. These materials form layered structures in which the different *X*-*M*-*X* layers are held together by weak van der Waals forces [20–26]. Yi Li [27] studied that the adsorption energy of COF_2_ on Ni-MoS_2_ was better than CF_4_, and Ni-MoS_2_ acted as electron donor and obvious charge transfer was observed. Soumyajyoti Haldar [28] reported that structural, electronic, and magnetic properties of atomic scale defects in 2D transition metal dichalcogenides MX_2_, and different vacancy had a great effect on different 2D dichalcogenides MX_2_, it is likely that band gap, density of states, some properties, and so on. Janghwan Cha [29] used different functionals to show the relatively binding energies about gas molecule and MoX_2_. The optPBE-vdW functionals showed relatively large binding energies. Furthermore, the TMDs are promising materials to realize gas sensors, so we study the effect of many defects on MoX_2_(X=S, Se, Te) for structure, band gap [30–32], adsorption energy, charge transfer, etc. This paper studied the interaction of methane with monolayer MoX_2_ by first-principle simulation (see Fig. 1). The green color ball is Mo atom, and the yellow color ball is X atom, the distance of d_1_ for S-S, Se-Se, and Te-Te is 3.190 Å, 3.332 Å, and 3.559 Å, respectively, the distance of d_2_ is the same as the three cases of d_1_. This work was based on DFT, and the adsorption energy, charge transfer, adsorption distance, and density of states (DOS) of CH_4_ gas molecule on MoX_2_ were studied.

## Method and Theory

A 4 × 4 supercell of MoX_2_ (32 X atoms and 16 Mo atoms) and CH_4_ gas molecule adsorbed onto it was built in Materials studio [33–36]. DMol^3^ [37] software was used for calculation. In this paper, the Perdew, Burke, and Ernzerhof (PBE) [38, 39] functions with generalized gradient approximation (GGA) were selected to describe the exchange energy Vxc. The Mo was generated in 4p^6^5s^1^4d^5^ configuration and another was used for the generation of the valence electrons of X. The Brillouin zone of MoX_2_ was sampled using a 6 × 6 × 1 k-point grid and Methfessel-Paxton smearing of 0.01 Ry. The cutoff energy was 340 eV with self-consistence-field (SCF) converged of 1.0 × 10^−5^ eV. All the atomic structures were relaxed until the maximum displacement tolerance of 0.001 Å and maximum force tolerance of 0.03 eV/Å [40, 41].

We calculated the adsorption energy (*E*_ad_) in the adsorbed systems, which was defined in the following equation:

$$ {E}_{\mathrm{a}}={{E_{\mathrm{MoX}2+\mathrm{CH}4\ \mathrm{gas}}}_{\mathrm{m}}}_{\mathrm{olecule}}-\left({E}_{\mathrm{MoX}2}+{E}_{\mathrm{CH}4\ \mathrm{gas}\ \mathrm{molecule}}\right) $$

Where, *E*_MoX2 + CH4 gas molecule,_ *E*_MoX2_ and E_CH4 gas molecule_ represent the energies of the monolayer MoX_2_ adsorbed system, monolayer MoX_2_, and a CH_4_ gas molecule, respectively. All energies achieve the best optimization after structural optimization. We used Mulliken’s population analysis to study the charge transfer.

## Results and Discussion

**Fig. 2:**
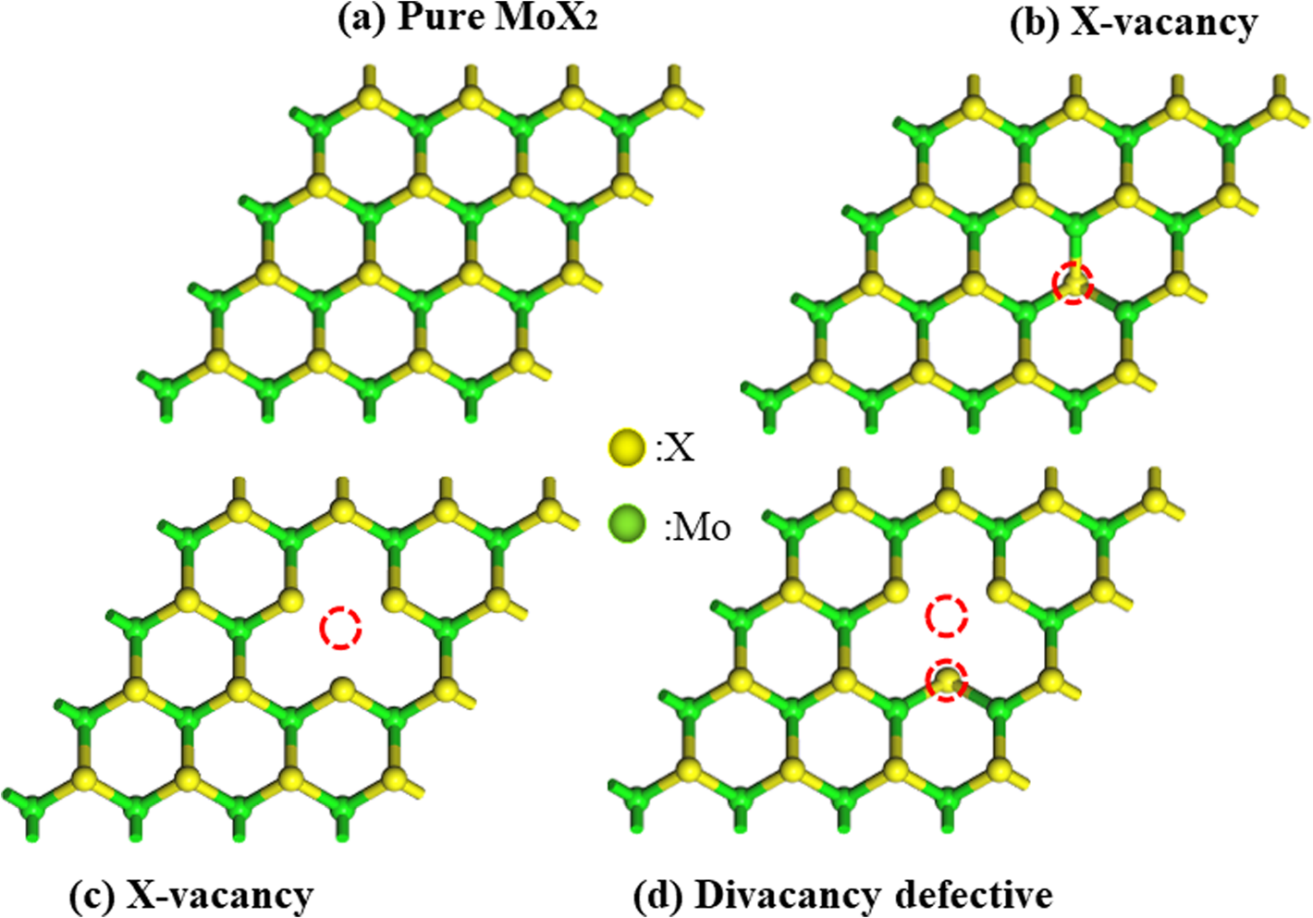
Top view of MoX_2_ with **a** pure MoX_2_, **b** S vacancy, **c** Mo vacancy, and **d** Divacancy. Green and yellow balls represent Mo and X(S, Se, Te) atoms, respectively.

Firstly, we discussed the geometric and electric structures of the four MoX_2_ substrates (ee in Fig. 2). The bond length of Mo-S, Mo-Se, and Mo-Te were 2.426 Å, 2.560 Å, and 2.759 Å, which were in good agreement with experimental value of 2.410 Å (MoS_2_) [42, 43], 2.570 Å (MoSe_2_) [44] and 2.764 Å (MoTe_2_) [45], the four structures MoX_2_ were in this paper, pristine MoX_2_, MV_X_(one X atom vacancy), MV_Mo_(one Mo atom vacancy), and MV_D_(one X atom and one Mo atom vacancy) respectively. Full structural relaxation showed that the stretching X-Mo bond length from 2.420 Å to 2.394 Å (MV_S_), 2.420 Å to 2.398 Å (MV_Mo_), and the main reason was that the absence of atoms enhanced the interaction between the adjacent Mo atoms and other S atoms, the chemical bond became stronger and the bond length became shorter.

**Fig. 3:**
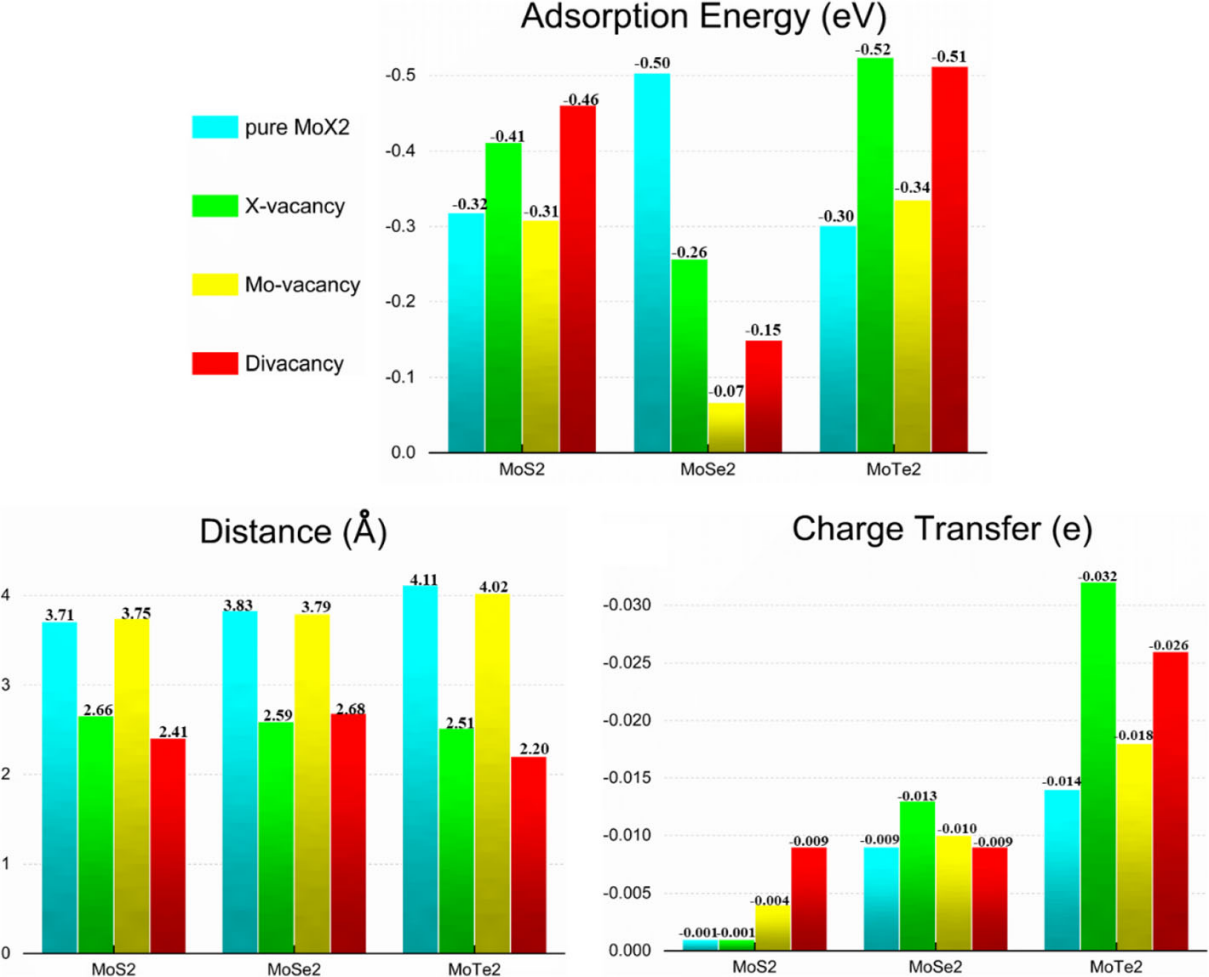
Adsorption energies, shortest atomic distances between molecule and MoX_2_, and charge transfers

Figure 3a–c displayed the calculated adsorption energy, charge transfer, and adsorption distance of CH_4_/MoX_2_ system. Before the adsorption, the distance between the CH_4_ gas molecules and the molybdenum disulfide was 3.6 Å. The CH_4_ gas molecule obtained about − 0.001 e to − 0.009 e from the four systems of MoS_2_ sheet, − 0.009 e to − 0.013 e from the four systems of MoSe_2_ sheet and − 0.014 e to − 0.032 e from the four systems of MoTe_2_ sheet, respectively, which means that CH_4_ acted as an acceptor. Inclusion of the van der Waals correction enhances the adsorption energies of CH_4_ gas molecule by − 0.31 eV to − 0.46 eV on the four systems of MoS_2_ systems, by − 0.07 eV to − 0.50 eV on the four systems of MoSe_2_ systems, and by − 0.30 eV to − 0.52 eV on the four systems of MoTe_2_ system, and 0.01 eV was usually thought within the error range. It was obvious that the adsorption distance was the shortest in the case of S atom defects and divacancy defects. To sum up the above data, we saw that the adsorption effect was the best under the condition of divacancy defected.

### Adsorption of CH_4_ Gas Molecule on Monolayer MoS_2_

**Fig. 4:**
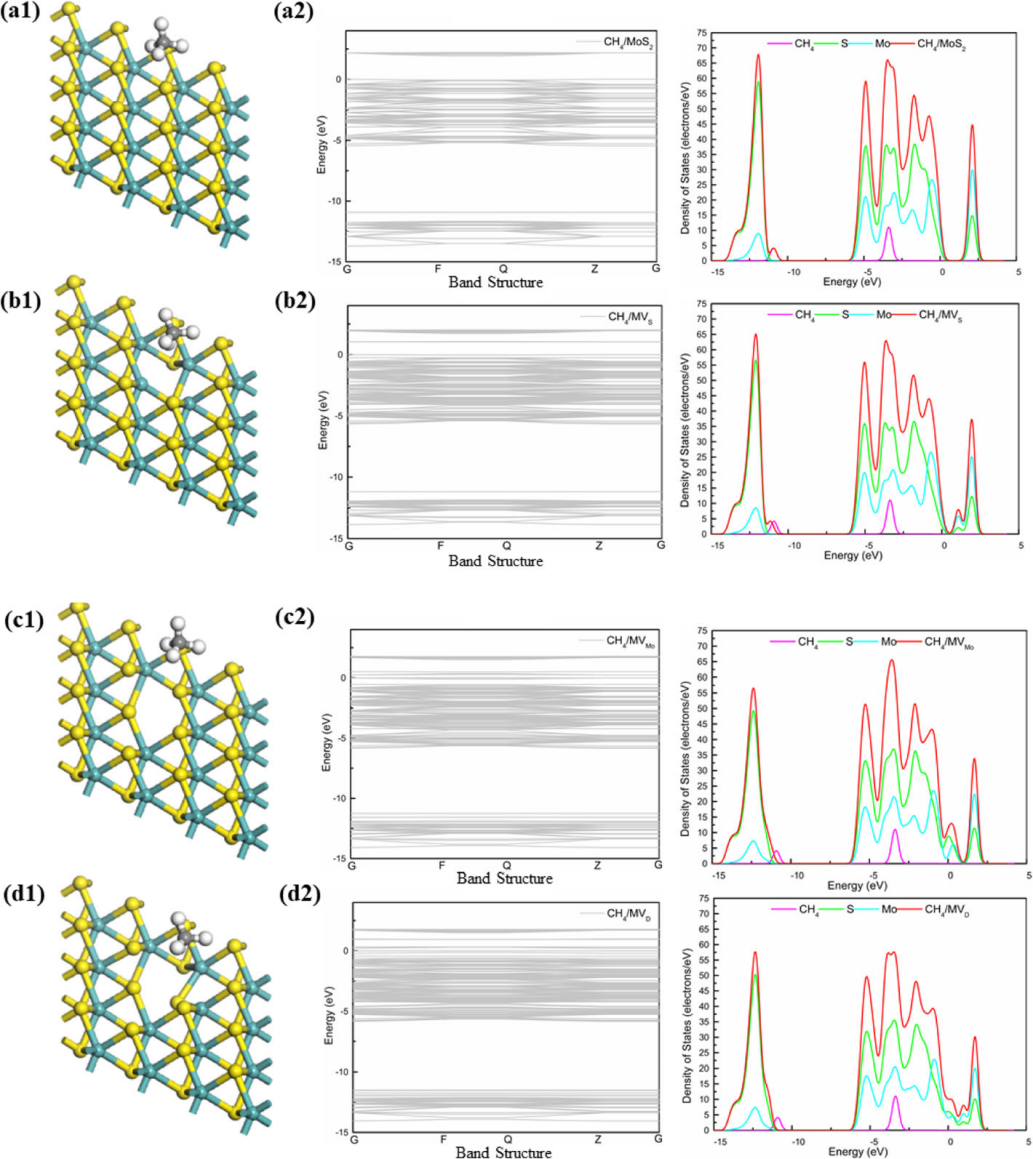
The structure and DOS of CH_4_ gas molecule on four systems (MoS_2_, MV_S_, MV_Mo_, and MV_D_)

In order to have a clear understanding about the bonding mechanism of CH_4_ gas molecule on pure and defected MoS_2_ (including MV_s_, MV_Mo,_ and MV_D_), we analyzed the corresponding density of states (DOS) for adsorbed CH_4_ gas molecule in adsorption structures. Comparing four systems, the adsorption effect of CH_4_ gas molecule on pure and defected MoS_2_ (including MV_s_, MV_Mo_, and MV_D_) were further investigated. The DOS (Fig. 4) showed that there was a certain change in the vicinity of the Fermi level, which was the same as the general DOS form. The energy band gap of four systems was observed along the gamma point (G) noticed to be 1.940 eV (MoS_2_), 1.038 eV (MV_S_), 0.234 eV (MV_Mo_), and 0.209 eV (MV_D_). Moreover, the observed energy band gap of MoS_2_ nanosheet was in good agreement with other reported theoretical work (1.78 eV [39], 1.80 eV [40]) and experimental work (1.90 eV [41], 1.98 eV [42]). Meantime, monolayers MoS_2_ had five peak values, the peak was − 12.2 eV, − 5 eV, − 4 eV, − 2 eV, and − 1 eV which were ascribed to the S atom in MoS_2_ and the Mo atom in MoS_2_. However, the DOS of four systems (Fig. 4) showed that the electronic level of CH_4_ gas molecule has a peak for about − 3 eV which was closed to Fermi level. It was contributed to the conduction band in the system and affects the conductivity of the system. Comparing four systems, the peak of − 12.5 eV MVs was obviously much lower than MoS_2_ because of the defect of the S atom in the MoS_2_. And the defects of the Mo atom do not have much effect; however, the contribution at the conduction zone was still decreasing. As shown in Fig. 3 b, obviously, the band around the 0 eV was getting smaller and smaller, and the curve was more and more stable. In summary, there was no bond between CH_4_ gas molecule and MoS_2_, and the electron transfer and adsorption energy were small, and the adsorption was not very strong, which was obviously physical adsorption.

### Adsorption of CH_4_ Gas Molecule on Monolayer MoSe_2_

**Fig. 5:**
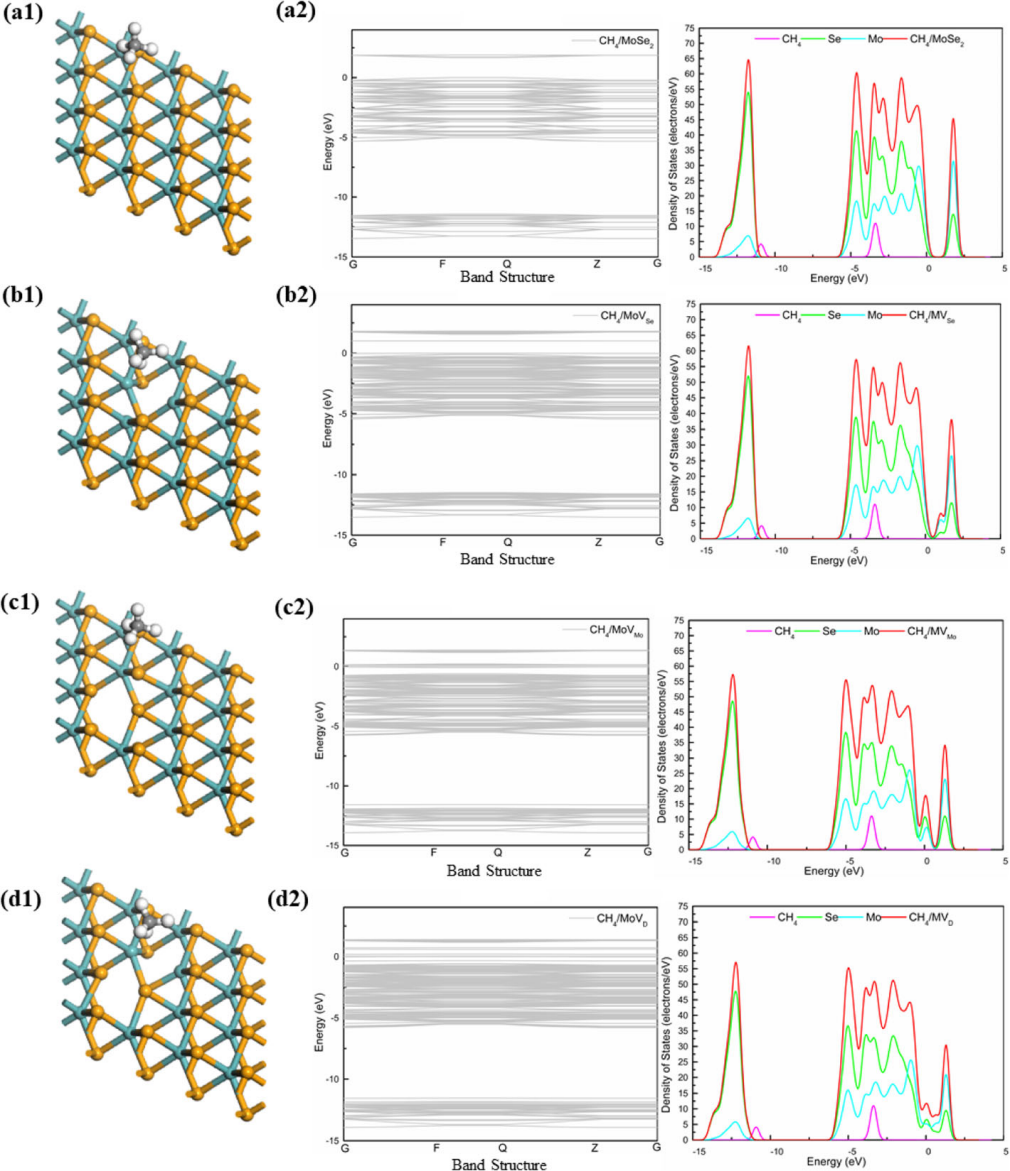
The structure and DOS of CH_4_ gas molecule on four systems (MoSe_2_, MV_Se_, MV_Mo_, and MV_D_)

We studied the adsorption of CH_4_ gas molecule on four systems of MoSe_2_, it could be seen from the DOS (Fig. 5) that the electron energy levels of CH_4_ gas molecule in the four adsorption orientations were close to the Fermi level, which had a certain influence on the conductivity of the system, and the band gap system was so small, same as adsorption of MoS_2_. Meantime, the DOS (Fig. 5) also showed that the Se atoms in MoSe_2_ had five peak values, the peak was − 12 eV, − 5 eV, − 4 eV, − 3 eV, and − 2 eV, the Mo atom in MoSe_2_ had overlapping peaks at about 0.5 eV and 2 eV. Compared with MoS_2_, Se contributed more to the system than S in MoS_2_ below the fermi level, and the energy band gaps of four systems were observed along the gamma point (G) that was noticed to be 1.680 eV (MoSe_2_), 1.005 eV (MV_Se_), 0.094 eV (MV_Mo_), and 0.024 eV(MV_D_). The band was narrower and more stable around the 0 eV. Therefore, it could be confirmed that the adsorption properties and the CH_4_ gas molecule on the four systems were physisorption.

### Adsorption of CH_4_ Gas Molecule on Monolayer MoTe_2_

**Fig. 6:**
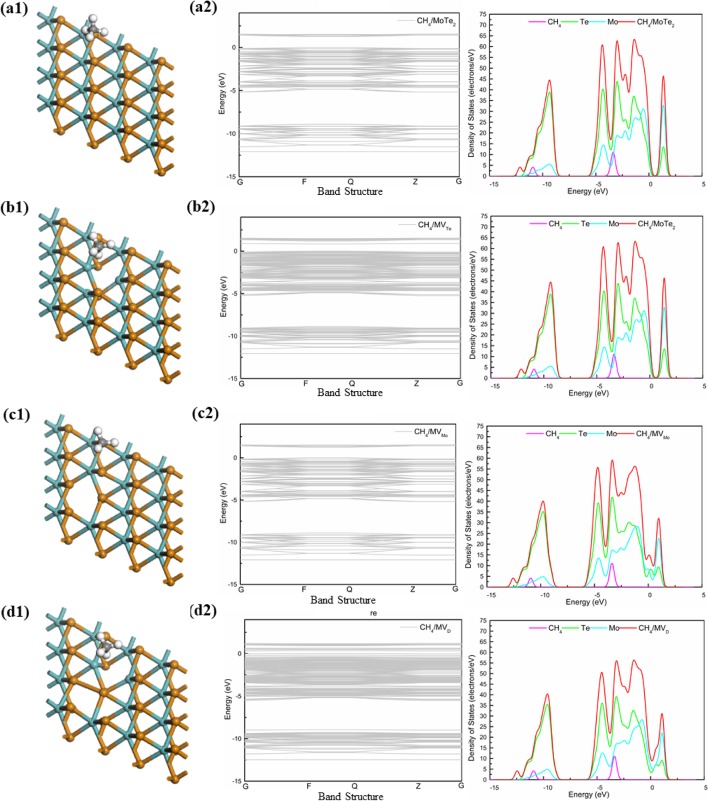
The structure and DOS of CH_4_ gas molecule on four systems (MoTe_2_, MV_Te_, MV_Mo_, and MV_D_)

We studied the adsorption of CH_4_ gas molecule on four systems of MoTe_2_, the DOS (Fig. 6) of CH_4_ gas molecule on the MoTe_2_ were analyzed. As shown in Fig. 6, the electronic levels of CH_4_ in the four MoTe_2_ systems were short with CH_4_/MoS_2_ systems and CH_4_/MoSe_2_ systems, and the energy band gap of four systems were observed along the gamma point (G) was noticed to be 1.261 eV (MoTe_2_), 0.852 eV (MV_Te_), 0 eV (MV_Mo_), and 0.316 eV (MV_D_). One of the strangest things of all was the defect of the Mo atom, which allowed the system to be transformed from semiconductor to metal. Meantime, the DOS (Fig. 6) also showed that the Te atoms in MoTe_2_ had four peaks value, the peak was − 10 eV, − 5 eV, − 3 eV, and − 1 eV and the Mo atom in MoSe_2_ had overlapping peaks at about 1 eV.

In general, on the basis of the adsorption behaviors of CH_4_ gas molecule in different systems, the CH_4_ gas molecule adsorbed by the MV_X_ could have two peaks near the Fermi level. The DOS between the two spikes was not zero but very wide, which reflected the strong covalent property of the system. To summarize all the data, the MV_Te_ might become an ideal sensing material for the detection of CH_4_ gas molecule.

## Conclusions

We carried out density-functional-GGA studies to study the interaction of an isolated CH_4_ gas molecule on MoX_2_ (X=S, Se, Te). The results indicated that the different defects changed the electrical properties of MoX_2_ greatly, and our results revealed a weak interaction between the CH_4_ gas molecules and MoX_2_ monolayer, which indicated the physical nature of the adsorption. The total electron density plots confirmed the physisorption of gas molecules on the MoX_2_ surface, as the material weakly interacts with the CH_4_ gas molecules without the formation of covalent bonds at the interface region. Furthermore, the structure of MV_D_ has a good band gap, semiconductor property, the best adsorption energy, and the stronger charge transfer for the CH_4_ gas molecule. Besides, the electronic band structures of the sensing system were altered upon the adsorption of gas molecules. MoTe_2_ had the highest adsorption energy (− 0.51 eV), the shortest intermolecular distance (2.20 Å), and the higher charge transfer (− 0.026 e). At last from the analysis of these three materials, it could be seen that MV_D_ (MoTe_2_) had the best adsorption effect on CH_4_ gas molecule. The calculated results thus suggested a theoretical basis for the potential application of MV_D_(MoTe_2_) monolayers in the CH_4_ based gas sensor devices.

## Data Availability

All data are fully available without restriction.

## Abbreviations

CH4: Methane
DOS: Density of states
Ea: Adsorption energy

## References

[1] Wetchakun K, Samerjai T, Tamaekong N (2011). Semiconducting metal oxides as sensors for environmentally hazardous gases [J]. Sens Actuators B.

[2] Abbasi A, Sardroodi JJ (2016). N-doped TiO_2_ anatase nanoparticles as a highly sensitive gas sensor for NO_2_ detection: insights from DFT computations [J]. Environ Sci: Nano.

[3] Ueda T, Bhuiyan MMH, Norimatsu H (2008). Development of carbon nanotube-based gas sensors for NOx gas detection working at low temperature [J]. Physica E: Low-dimensional Systems and Nanostructures.

[4] Abbasi A, Sardroodi JJ (2016) Theoretical study of the adsorption of NOx on TiO2/MoS2 nanocomposites: a comparison between undoped and N-doped nanocomposites[J]. J Nanostruct Chem

[5] Abbasi T, Abbasi SA (2011). ‘Renewable’hydrogen: prospects and challenges [J]. Renew Sustain Energy Rev.

[6] Reddy RG (2006). Fuel cell and hydrogen economy [J]. J Mater Eng Perform.

[7] Cheng X, Shi Z, Glass N (2007). A review of PEM hydrogen fuel cell contamination: Impacts, mechanisms, and mitigation [J]. J Power Sources.

[8] Novoselov KS, Morozov SV, Mohinddin TMG (2010). Electronic properties of graphene [J]. Physica Status Solidi.

[9] Jose D, Datta A (2013). Structures and chemical properties of silicene: unlike graphene [J]. Acc Chem Res.

[10] Hasan MZ, Kane CL (2010). Colloquium: topological insulators [J]. Rev Mod Phys.

[11] Stankovich S, Dikin DA, Dommett GHB (2006). Graphene-based composite materials [J]. Nature.

[12] Nijamudheen A, Bhattacharjee R, Choudhury S (2015). Electronic and chemical properties of germanene: the crucial role of buckling [J]. J Phys Chem C.

[13] Chowdhury C, Jahiruddin S, Datta A (2016). Pseudo-Jahn–Teller distortion in two-dimensional phosphorus: origin of black and blue phases of phosphorene and band gap modulation by molecular charge transfer [J]. J Phys Chem Lett.

[14] Bhattacharjee R, Majumder T, Datta A (2019) Analysis of pseudo jahn–teller distortion based on natural bond orbital theory: case study for silicene [J]. J Comb Chem 10.1002/jcc.25815 30854679

[15] Ni J, Yang B, Jia F (2018). Theoretical investigation of the sensing mechanism of the pure graphene and AL, B, N, P doped mono-vacancy graphene-based methane[J]. Chem Phys Lett.

[16] Qiao X, Chao Z, Shao Q et al Structural characterization of corn stover lignin after hydrogen peroxide presoaking prior to ammonia fiber expansion pretreatment [J]. Energy Fuels, 2018: acs. energyfuels. 8b00951

[17] Zhao C, Yan C, Ma Z (2017). Optimization of liquid ammonia pretreatment conditions for maximizing sugar release from giant reed (Arundo donax L) [J]. Biomass Bioenergy.

[18] Zhao C, Shao Q, Ma Z (2016). Physical and chemical characterizations of corn stalk resulting from hydrogen peroxide presoaking prior to ammonia fiber expansion pretreatment [J]. Ind Crops Prod.

[19] Zhao C, Qiao X, Cao Y (2017). Application of hydrogen peroxide presoaking prior to ammonia fiber expansion pretreatment of energy crops. Fuel.

[20] Mak KF, Lee C, Hone J (2010). Atomically thin MoS 2: a new direct-gap semiconductor [J]. Phys Rev Lett.

[21] Pan H, Zhang YW (2012). Edge-dependent structural, electronic and magnetic properties of MoS_2_ nanoribbons [J]. J Math Chem.

[22] Ataca C, Sahin H, Ciraci S (2012). Stable, single-layer MX_2_ transition-metal oxides and dichalcogenides in a honeycomb-like structure [J]. J Physic Chem C.

[23] Pan H, Zhang YW (2012). Tuning the electronic and magnetic properties of MoS2 nanoribbons by strain engineering [J]. J Phys Chem C.

[24] Yin Z, Li H, Li H (2011). Single-layer MoS_2_ phototransistors [J]. ACS Nano.

[25] Frindt RF (1966). Single crystals of MoS_2_ several molecular layers thick [J]. J App Phys.

[26] Joensen P, Frindt RF, Morrison SR (1986). Single-layer MoS_2_ [J]. Mater Res Bull.

[27] Li Y, Zhang X, Chen D (2018). Adsorption behavior of COF_2_ and CF_4_ gas on the MoS_2_ monolayer doped with Ni: A first-principles study [J]. App Surf Sci.

[28] Haldar S, Vovusha H, Yadav MK (2015). Systematic study of structural, electronic, and optical properties of atomic-scale defects in the two-dimensional transition metal dichalcogenides MX_2_ (M= Mo, W; X= S, Se, Te) [J]. Phys Rev B.

[29] Cha J, Sung D, Min KA (2018). Van der Waals density functional theory study of molecular adsorbates on MoX_2_ (X= S, Se or Te) [J]. J Korean Phys Soc.

[30] Li H, Huang M, Cao G (2016). Markedly different adsorption behaviors of gas molecules on defective monolayer MoS_2_: a first-principles study [J]. Phys Chem Chem Phys.

[31] Lee C, Hone J, Shan J (2010). Atomically 392 Thin MoS_2_: A new direct-gap semiconductor [J]. Phys. Rev. Lett.

[32] Ma D, Wang Q, Li T (2016). Repairing sulfur vacancies in the MoS_2_ monolayer by using CO, NO and NO_2_ molecules [J]. J Mat Chem C.

[33] Shokri A, Salami N (2016). Gas sensor based on MoS2 monolayer[J]. Sens Actuators B.

[34] Yue Q, Shao Z, Chang S (2013). Adsorption of gas molecules on monolayer MoS 2 and effect of applied electric field [J]. Nanoscale Res Lett.

[35] Zhao S, Xue J, Kang W (2014). Gas adsorption on MoS2 monolayer from first-principles calculations [J]. Chem Physics Lett.

[36] Ray SJ (2016). First-principles study of MoS2, phosphorene and graphene based single electron transistor for gas sensing applications [J]. Sens Actuators B: Chem.

[37] O.-T W J. Molecular mechanics: by U. Burkert and N. L. Allinger, American Chemical Society, Washington. Journal of Molecular Structure Theochem [J],109(3–4) (1984) 401-401.

[38] Kohn W, Sham LJ (1965). Self-consistent equations including exchange and correlation effects [J]. Phys Rev.

[39] Perdew JP (1997). JP Perdew, K. Burke, and M. Ernzerhof, Phys. Rev. Lett. 78, 1396 (1997) [J]. Phys. Rev. Lett..

[40] Monkhorst HJ, Pack JD (1976). Special points for Brillouin-zone integrations [J]. Phys Rev B.

[41] Becke AD (1993). A new mixing of Hartree–Fock and local density-functional theories [J]. J Chem Phys.

[42] Komsa HP, Krasheninnikov AV (2015). Native defects in bulk and monolayer MoS 2 from first principles [J]. Phys Rev B.

[43] Zahid F, Liu L, Zhu Y (2013). A generic tight-binding model for monolayer, bilayer and bulk MoS2 [J]. Aip Advances.

[44] Sharma M, Jamdagni P, Kumar A (2016). Interactions of gas molecules with monolayer MoSe2: A first principle study [C]//AIP Conference Proceedings. AIP Publishing.

[45] Fan Z, Wei-Bing Z, Bi-Yu T (2015). Electronic structures and elastic properties of monolayer and bilayer transition metal dichalcogenides MX2 (M= Mo, W; X= O, S, Se, Te): a comparative first-principles study [J]. Chinese Physics B.

